# Strategies for detecting and identifying biological signals amidst the variation commonly found in RNA sequencing data

**DOI:** 10.1186/s12864-021-07563-9

**Published:** 2021-05-03

**Authors:** William W. Wilfinger, Robert Miller, Hamid R. Eghbalnia, Karol Mackey, Piotr Chomczynski

**Affiliations:** 1 Molecular Research Center, Inc., Cincinnati, USA; 2 Robert Miller Enterprises, LLC, Cincinnati, USA; 3 University of Wisconsin-Madison, Madison, USA; 4 University of Cincinnati, Cincinnati, USA

**Keywords:** Scaling, Rank-order, Trendline, Biological variability, Biological pathway analysis, RNA sequencing, STRING-db, Minimum value adjustment, White blood cells

## Abstract

**Background:**

RNA sequencing analysis focus on the detection of differential gene expression changes that meet a two-fold minimum change between groups. The variability present in RNA sequencing data may obscure the detection of valuable information when specific genes within certain samples display large expression variability. This paper develops methods that apply variance and dispersion estimates to intra-group data to identify genes with expression values that diverge from the group envelope. STRING database analysis of the identified genes characterize gene affiliations involved in physiological regulatory networks that contribute to biological variability. Individuals with divergent gene groupings within network pathways can thereby be identified and judiciously evaluated prior to standard differential analysis.

**Results:**

A three-step process is presented for evaluating biological variability within a group in RNA sequencing data in which gene counts were: (1) scaled to minimize heteroscedasticity; (2) rank-ordered to detect potentially divergent “trendlines” for every gene in the data set; and (3) tested with the STRING database to identify statistically significant pathway associations among the genes displaying marked trendline variability and dispersion. This approach was used to identify the “trendline” profile of every gene in three test data sets. Control data from an in-house data set and two archived samples revealed that 65–70% of the sequenced genes displayed trendlines with minimal variation and dispersion across the sample group after rank-ordering the samples; this is referred to as a linear trendline. Smaller subsets of genes within the three data sets displayed markedly skewed trendlines, wide dispersion and variability. STRING database analysis of these genes identified interferon-mediated response networks in 11–20% of the individuals sampled at the time of blood collection. For example, in the three control data sets, 14 to 26 genes in the defense response to virus pathway were identified in 7 individuals at false discovery rates ≤1.92 E-15.

**Conclusions:**

This analysis provides a rationale for identifying and characterizing notable gene expression variability within a study group. The identification of highly variable genes and their network associations within specific individuals empowers more judicious inspection of the sample group prior to differential gene expression analysis.

**Supplementary Information:**

The online version contains supplementary material available at 10.1186/s12864-021-07563-9.

## Background

A major goal of RNA-seq studies is to improve and extend our understanding of gene expression responses amidst the challenging variability commonly found in sequencing data. Although numerous factors are known to affect sequencing results such as the reference genome, the read processing pipeline, internal references, read fragment size, and the selected data analysis algorithms, among others [1], thus far it has been difficult to discern how these sequencing procedures combined with intrinsic biological variability might impact differential analysis. For example, many software packages commonly employ different normalization procedures that are designed to mitigate read count variability; however, these strategies are known to yield dissimilar differential expression analysis results [2–6]. Biological variation is considered to be larger than technical variation [3, 6–8], but the biological implications associated with read count normalization are not well-understood. Previous studies have suggested that increasing the sequencing depth (read coverage) and/or the number of biological replicates generally improves estimates of biological variation [6–8].

Conclusions relating to biological variation are usually based on Analysis of Variance (ANOVA) Sums of Squares estimations. Although increasing the level of replication may increase the Between Sums of Square difference and provide a more definitive statistical conclusion about an identified biological response (e.g. larger F-value), an increase in the Sums of Squares does not identify the factor(s) contributing to the variability. More broadly untangling the impact of variability on each step of the RNA-seq pipeline is difficult. One must identify specific sources of biological variability in the data set and consider how the normalization process impacts the overall results. This problem becomes increasingly difficult to resolve in samples in which cell number and cell type fluctuate significantly. Identifying and quantifying significant variability within RNA sequencing data sets would provide information that would be very useful for evaluating the robustness of computational steps, for example, devising and evaluating methodologies for determining how normalization protocols impact technical and biological variation.

Van den Berg et al. [9] have employed various scaling strategies to their metabolomics data and examined their usefulness in categorizing the relative importance of various metabolites identified in these studies. They determined that scaling normalizations performed better than other strategies because they removed the dependence of the metabolites initial ranking based on the magnitude of a quantitative response. The scaled metabolites were evaluated in relation to their sample-to-sample response range which also reduced the heteroscedasticity (mean and variance dispersion) within the data set. Since these data sets were qualitatively similar to the data obtained in RNA sequencing studies, we applied an approach similar to scaling normalization to evaluate RNA sequencing results.

Blood from 35 healthy adults was extracted and processed for RNA sequencing [10, 11]. The read counts were scaled to establish a uniform starting point across all genes and rank-ordered to characterize gene expression in the sample group as a “trendline” pattern for each gene. Excel-based tools were employed to analyze and catalogue the resulting gene trendlines [12]. Utilizing trendline analysis, we determined that 65–70% of the genes in our control data set follow a linear relationship with minimal variance when the genes were scaled and rank-ordered. However, other genes that did not follow this linear profile displayed markedly higher levels of dispersion and variability that diverged significantly from the genes in a normally distributed control sample. We identified standard statistical measures that characterize and catalogue these different trendlines and utilized this information to identify factors that may contribute to this heightened biological variability. When genes displaying the most variable and dispersed trendline expression patterns were evaluated with the STRING database [13–15], distinct biological regulatory pathways were identified in some individuals, thereby providing an explanation for some of the variability in the sample group.

We also demonstrate that the scaling normalization strategy employed in our study reduced gene expression heteroscedasticity within three different control data sets as previously demonstrated by van den Berg et al. [9]. Scaling adjustments in conjunction with rank-order analysis clarify and extend the analysis of inter-individual variations relating to differential gene expression previously described by Whitney et al. [16], Savelyeva et al. [17], Preininger et al. [18] and Jaffe et al. [19] to within-the-group analysis. STRING-db analysis of genes displaying the most variable and dispersed trendlines revealed that 11–20% of the individuals in our control sample and two archived control data sets, identified a prominent network of interferon-stimulated genes. The interferon-induced genes identified in this analysis play a pivotal regulatory role in three Gene Ontology pathways [20–22] that include response to virus, defense response to virus and the type I-interferon signaling/regulatory response pathways. The evaluation of gene trendline responses within a group and across individuals identifies sources of previously unrecognized biological variability that now can be detected and appraised. This method of analysis can be applied to archived RNA sequencing data to detect previously unrecognized sources of biological variability that may have impacted differential analysis and physiological conclusions. The methods outlined in this report will be useful in identifying within group variability commonly found in RNA sequencing data sets and when employed in conjunction with established data processing pipelines, they are likely to improve the robustness of these studies.

## Results

### Rank-ordering RNA sequencing counts graphically portrays the impact of sample dispersion on gene trendline profiles

**Fig. 1:**
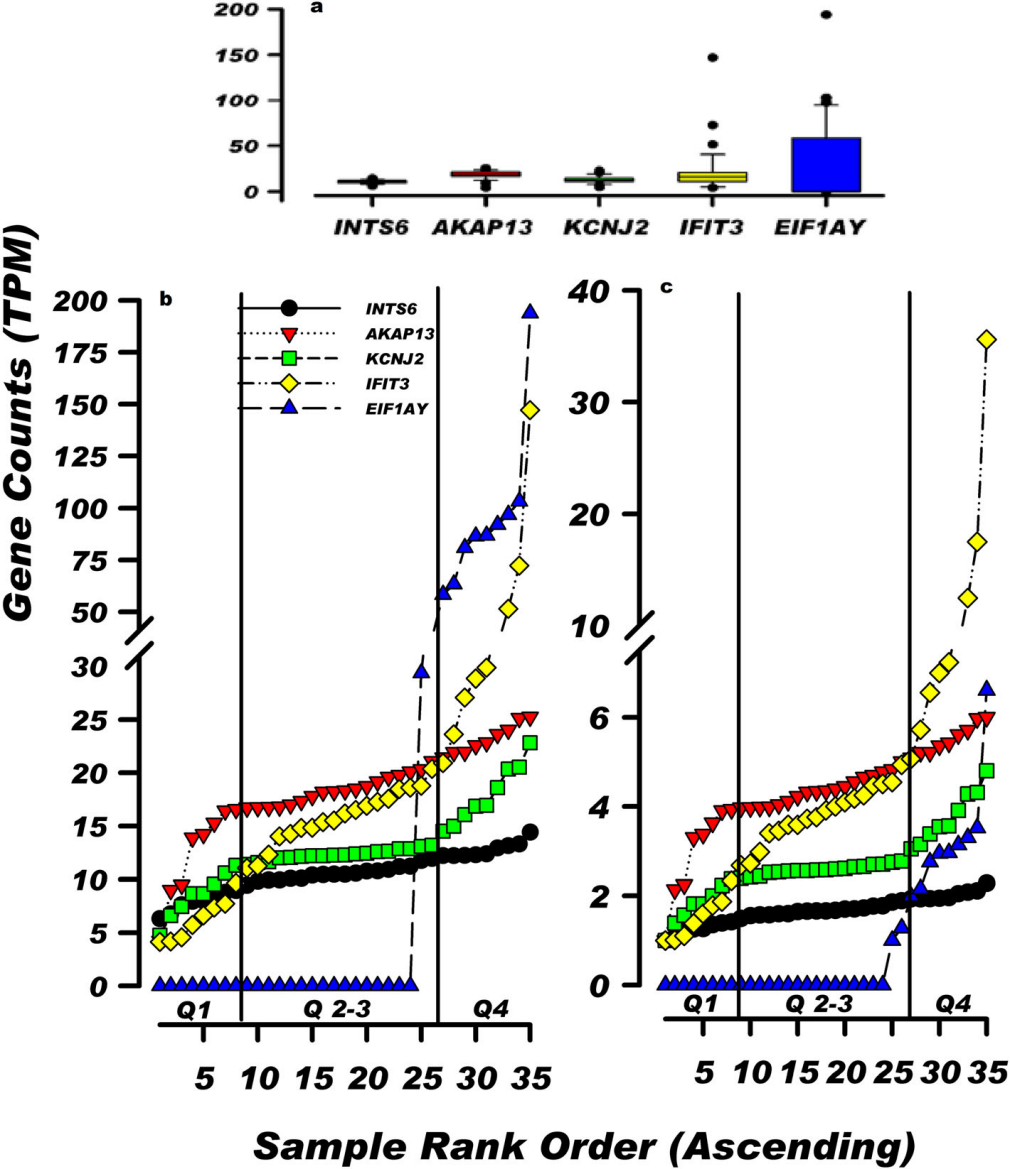
Rank-ordering RNA sequencing counts identifies individuals displaying gene count divergence. **a** Box plots of sequencing counts for five genes INTS6, AKAP13, KCNJ2, IFIT3 and EIF1AY depicting increasing levels of sample dispersion with computed coefficient of variation values ranging from 17.9 to 171.2% of the unadjusted TPM gene counts (Mean ± 1SD). Box boundaries exclude individuals in the first and fourth quartile for each gene. **b** Rank-ordering the unadjusted counts of 35 individuals delineates different gene trendline patterns for the five genes. Gene rank-order position is established in relation to the gene expression level for an individual gene within the sample group, therefore the ranking order does not identify the same individual at each position along the various gene trendlines since the relative level of gene expression for an individual changes across genes. **c** Minimum Value Adjusted (MVA) gene counts significantly improve count heteroscedasticity (5-fold scale reduction) without altering the incremental trendline profiles within the sample group. Rank-order analysis extends the descriptive sample information available from a box plot by: defining the number of data points within the sample that deviate from the count level in the 2nd and 3rd quartiles; identifying their inflection point(s) and providing an estimate of the relative change in gene expression based on the computed slope ratio change. Black vertical lines identify quartiles 1, 2–3 and 4. See Additional file 1 for a more detailed discussion

DeSeq-normalized TPM (Transcripts Per kilobase Million) gene counts for 35 individuals were processed through our pipeline [23] and the count data were rank-ordered to construct a unique trendline for each gene. Figure 1a depicts a box plot of data for five example genes displaying increasing variance where the box boundaries identify gene counts in the 2^ed^ and 3rd quartiles (25th–75th percentile). The breadth of the box illustrates the degree of count dispersion across the 35 data points for each gene. The mean for the INTS6 gene is 10.52 ± 1.88 (1 SD) counts and plotting the counts for the 35 samples in ascending rank-order created a linear INTS6 trendline as illustrated in Fig. 1b. A coefficient of variation (CV) of 17.9% and the coefficient of determination (R^2^) of 0.9498 further supports the linear profile of the INTS6 trendline. This trendline profile was identical to the pattern obtained when numbers were randomly selected from a normally distributed population within a defined range of values and rank-ordered (see Additional file 1 for a detailed discussion). Therefore, we conclude that genes displaying a linear trendline profile across a defined range of expression values represent a “normally distributed control envelope” grouping of expression values within the identified samplying window.

The mean counts for genes AKAP13 and KCNJ2 were 18.26 ± 4.47 and 12.88 ± 3.82, respectively (Fig. 1a). While these genes showed slightly more dispersion across the 35 samples (Panels  a and b, with CV values of 25.26 and 29.62% and R^2^ values of 0.8499 and 0.8418, respectively), rank-ordering the counts revealed more complex trendlines where the slope of the line for samples in quartiles 1 and/or 4 deviated from the slope of the line for samples in quartiles 2 plus 3 (Fig. 1, panel b).

The last two example genes, IFIT3 and EIF1AY, displayed much greater deviation from the linear trendline model (Fig. 1a; 21.96 ± 25.52 and 26.88 ± 46.03, respectively). The rank-ordered IFIT3 trendline depicted in Fig. 1b, identified individuals in quartile 4 with markedly different expression levels when compared to individuals in quartiles 1–3. The final example gene, EIF1AY, is located on the Y chromosome and is expressed only in males. The gene trendline in Fig. 1b, shows an expected bimodal pattern with samples 24–35 comprising the eleven males in the sample group. The R^2^ values for these two genes were 0.429 and 0.5923, respectively, which denotes a significant deviation from linearity (CV 116.18 and 171.24%, respectively).

These five example genes exhibit increasing degrees of gene expression variability among the individuals in quartiles 1 and 4. The observed trendline profiles illustrate how rank-ordering of RNA sequencing counts can identify marked changes in gene expression variability among some of the 8746 protein coding genes identified in our study. Based on linear regression analysis, 65–70% of the 8000 to 10,000 evaluated genes (3 data sets) displayed trendlines where the incremental difference in gene expression across the group followed a linear pattern resulting in R^2^ values that were ≥ 0.9 (e.g. INTS6, Fig. 1, panel b). Under ideal conditions with minimal within sample variation, one might expect all of the sequenced genes in the control sample to follow this linear pattern but this is not the case. Our subsequent analysis attempts to provide some explanation for the heightened variability noted for genes such as IFIT3 in Fig. 1.

Figure 1c depicts the Minimum Value Adjusted (MVA) TPM counts which substantially reduce the range of gene expression (e.g. > 5-fold decrease in scale); however, the unique incremental sample-to-sample gene expression relationship of the 35 rank-ordered samples was maintained irrespective of the trendline profile (Fig. 1, panels b vs. c). When the quartile slopes for individuals in quartiles 1 and/or 4 deviates from those in quartiles 2 plus 3, a “tailing” profile was established as illustrated by the genes depicted in panels b and c of Fig. 1. Due to random chance, it would be difficult and unlikely to find several hundred genes displaying 4–8 “outliers” in a common subset of 35 individuals. Furthermore, we will now demonstrate how these “tailing response” profiles, as illustrated for the IFIT3 gene, can be used to identify other genes sharing comparable trendline profiles, and thereby identify sources of biological variation among selected individuals in a sample group.

### Statistical characterization of trendline “tailing responses” identify gene pathway regulatory groupings that contribute to biological variability

After rank-ordering unadjusted and MVA gene counts to create gene trendlines, standard Excel functions were used to perform a variety of statistical calculations [12]. Mean and median calculations measure aspects of dispersion and skewness, standard deviation, range, and slope measure dispersion, and skewness measures the unevenness of dispersion. Ranking these statistical parameters characterizes the degree to which this dispersion impacts gene expression levels for various genes. Calculations were computed for each of the 8746 genes and the results were ranked in descending order (Additional file 2, sheet 6). The 300 genes displaying the largest numerical values for each calculation were subjected to STRING-db analysis and the identified genes were surveyed for pathway affiliations (Additional file 2, sheet 7). The results were summarized and presented in Additional file 4A and B.

The unadjusted and MVA gene counts identified Biological Gene Ontology (GO) pathways associated with cotranslational protein targeting to membrane (section 4A) or immune system process pathways (section 4B) when the largest means representing the various statistical calculations were evaluated for the two groups. The unadjusted mean counts identified gene pathway groupings having the largest relative gene expression levels. When the gene counts are scaled by MVA to reflect the sample-to-sample incremental changes of each gene, the resulting trendline means identified immune pathway classifications rather than the highly expressed genes associated with protein synthesis (Additional file 4, panel A vs. B). The identification of markedly different pathway affiliations following MVA is consistent with the findings reported by van den Berg et.al [9].. When the unadjusted gene counts were used for these calculations, parameters that measure the relative magnitude of the count, such as mean, standard deviation, maximum, median, quartile 1, quartile 3, slope etc. all select highly expressed genes in Biological GO pathways associated with protein synthesis and targeting proteins to different areas of the cell (Panel 4A vs 4B). However, when statistical parameters such as range/median, skewness and kurtosis were used that characterize the “tailedness” and the unevenness of sample dispersion, identical pathway results were obtained with either unadjusted or MVA counts (Panel 4A vs 4B). Therefore, the type of measurement used for gene trendline characterization prior to STRING-db analysis impacts pathway selection if the heteroscedastic nature of the raw counts was not addressed prior to pathway analysis.

Other statistical calculations that measure sample variability and trendline asymmetry such as coefficient of variation, maximum/minimum ratio, range/median, skewness, kurtosis, range/quartile 3, and R^2^ all identified immune-related GO pathways with FDR’s ranging from E-6 to E-32 (Panel 4B). The 300 genes displaying the largest range/Q3 (FDR = 6.22 E-32), range/median (FDR = 5.33 E-26) and kurtosis values (FDR = 6.85 E-27) detected the greatest trendline variability and had the smallest R^2^ values ranging from 0.2253 to 0.8754. These three statistical calculations selected trendline “tailing” patterns with the greatest fidelity that were similar to the profile previously depicted by the IFIT3 gene in Fig. 1c.

The statistical parameters depicted in file 4 illustrate that some measures identified a larger number of gene associations with lower False Discovery Rates (FDR) based on the observed “tailing” patterns. Range/Q3, range/median and kurtosis measures detected 122, 113 and 105 immune system process (GO:0002376) pathway genes, respectively. Although all three parameters demonstrated proficiency in selecting genes with “tailing” profiles, only 8 of the top 10 pathways were identical among the three calculations and 7–14% fewer total genes were identified when either kurtosis or range/median measures were employed. Although a variety of calculations can be used for identifying gene pathway affiliations in addition to range/Q3, range/median and kurtosis, the other parameters selected fewer genes, different rank-orders, and alternative pathways when these parameters were employed to identify gene affiliations based on gene trendline tailing response profiles (Additional file 4). Changes in the order of the top 10 identified pathways were impacted by the number of known genes in a designated pathway and the selected measure used to identify the pathway-related genes in the sample. For example, the identification of 50 genes in a pathway of 200 genes provides a lower FDR than the detection of 50 genes in a pathway containing 2000 genes.

The identification of the top 300 computed trendline values, as outlined above, was also used to evaluate gene groupings that were selected using various combinations of sample size (e.g. 250–450 genes) and statistical parameter groupings (combine 1–3 measures for pathway selection). STRING-db analysis of 250–300 genes based on trendline kurtosis estimates selected identical pathways (data not shown). Samples of 300 genes surveyed at various rank position locations, ranging from 1 to 6000, selected different GO pathways with lower FDR’s following STRING-db analysis. Sampling genes at lower gene rankings identified large pathways involved in cellular metabolism and function. These pathways involve thousands of genes and due to the size of the pathways much lower FDR’s were observed (e.g. FDR > E-15).

The application of the MVA scaling reduced heteroscedasticity as previously noted [9] while preserving important sample-to-sample incremental changes that contributed to the rank-ordered trendline profiles. In our sample of 35 individuals, MVA reduced Total Sums of square by 960-fold and Within Group Sums of Square by 303-fold (see Additional file 1). The various statistical parameters tested in our studies revealed that range/Q3, range/median and kurtosis were the most sensitive and robust parameters for identifying “tailedness” in unadjusted as well as MVA applications (Additional file 4B).

### Correlation analysis identifies genes displaying similar trendline profiles and regulatory pathway associations

**Fig. 2:**
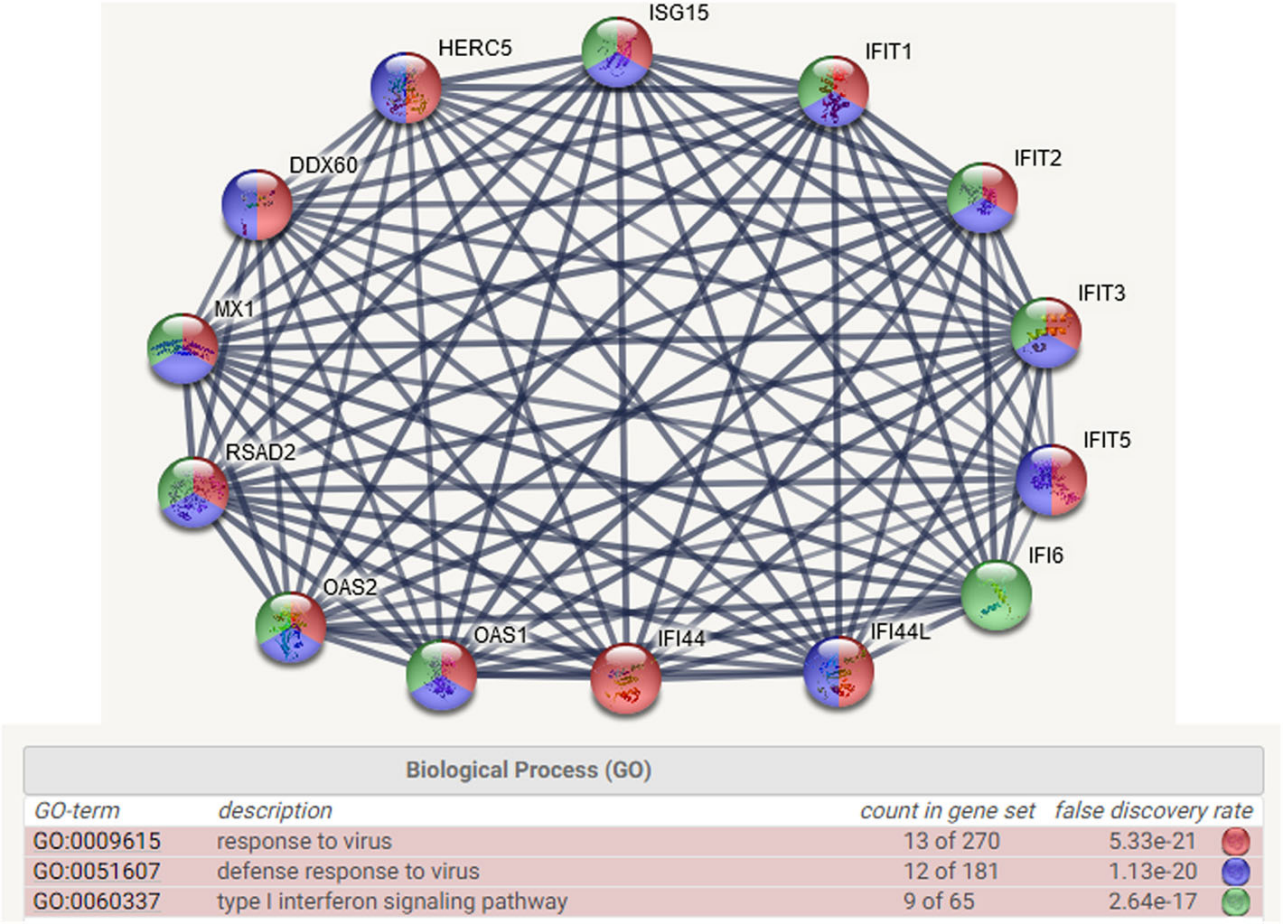
Listing of highly correlated genes identified by correlation analysis and their known integrated network affiliations within the immune system. STRING database analysis of the 13 genes found to be highly correlated (r ≥ 0.95725) with the IFIT3 gene. This regulatory cluster is associated with 24 GO pathways that are primarily involved in response to virus (red, GO:0009615), defense response to virus (blue, GO:0051607) and type 1 interferon signaling (green, GO:0060337). Eight of the highlighted genes (red, blue and green) form statistically significant groupings with False Discovery Rates ranging from E^− 17^ to E^− 21^ that may collectively integrate the activity of all three pathways

The previous analysis demonstrated that ranking certain statistical measures in a sample of 35 individuals identified genes with “tailed” trendlines and affiliated pathway groupings. To further evaluate this result, we employed correlation analysis to identity genes that might display similar associations to the trendline profiles previously noted for the IFIT3 gene (Fig. 1b and c). We used Excel to perform Pearson correlation analysis on the MVA counts of 8746 genes in our study [12]. To limit the size of the correlation matrix (> 78 × 10^6^ values) to a more discernable number of terms, estimated values for the highest correlation and anticorrelation range was used to provide a count of the number of genes displaying correlation values > or < input values and the number of genes assigned r values ≥ or ≤ the input terms were identified [12]. After the initial analysis, the input correlation values are adjusted up or down to limit the number of genes assigned to a smaller correlation subset matrix. Using this rationale, we identified a subset of 500 genes with correlation values ≥0.95725 or ≤ − 0.524674. Within this group of genes, the IFIT3 gene was positively correlated with the largest cluster of genes including IFIT1 and 12 other genes. STRING-db analysis indicated that these 14 genes were associated with 24 GO pathways containing multiple regulatory protein associations as depicted in Fig. 2. The top 3 GO pathways with FDR ≤ E-15 were GO:0009615, response to virus, 5.33 E-21; GO:0051607, defense response to virus, FDR 1.13 E-20 and GO:0060337, type 1 interferon signaling pathway, 2.64 E-17. The correlation results were identical when either the original counts or MVA counts were evaluated with an equivalent number of genes (i.e. 500). STRING-db analysis of the most highly correlated genes within the entire data set identified gene pathways that were activated in response to virus exposure.

**Fig. 3:**
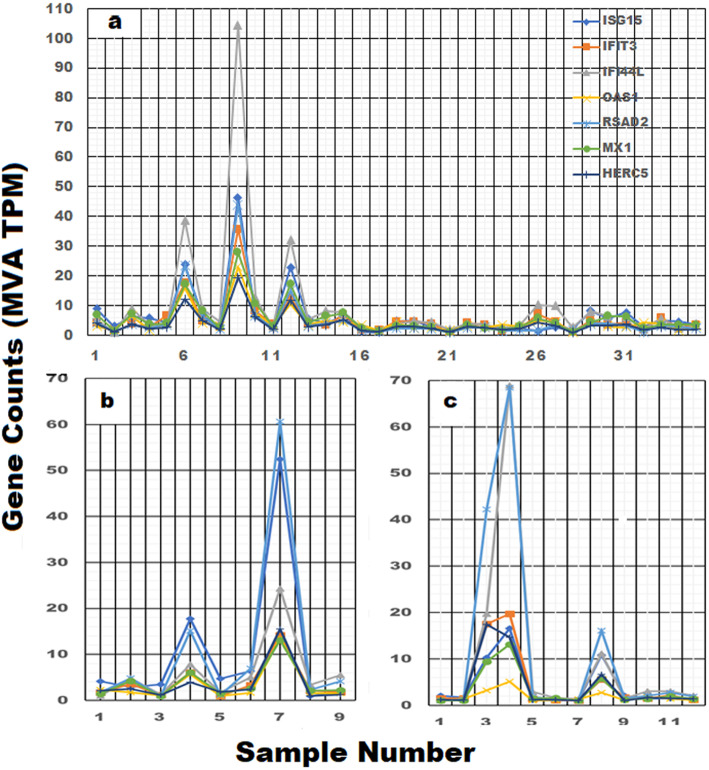
Highly correlated and functionally related gene networks are simultaneous elevated in specific individuals. Seven genes were selected from the highly correlated list of genes identified in Fig. 2 and their unranked expression profiles were plotted for the individuals in three different Control data sets (**a**, **b**, and **c**). In panel **a** (35 in house Controls), **b** (9 Controls, [24]) and **c** (12 Controls, [25]) the interferon induced IFI44L and ISG15 genes were specifically elevated in approximately 12% of the individuals (gene expression levels > 6-fold of baseline expression)

Based on the STRING-bd results presented in Fig. 2, 7 genes displaying two or more pathway affiliations were selected and their expression profiles were plotted in the 35 unranked control samples. The gene expression profiles for our control group and two additional archived control data sets are presented in Fig. 3. The average baseline expression level for most of these genes is ~ 5 counts, so gene expression levels of 30–110 counts represent markedly elevated levels of gene expression in certain individuals. Interferon induced IFI44L and ISG15 genes are markedly elevated in individuals 6, 9 and 12 in panel a, sample 7 in panel b and samples 3 and 4 in panel c, and the coordinated response is suggestive of individuals responding to the presence of a virus. It is important to emphasize that the elevated level of gene expression of these 7 genes is confined to specific individuals in the sample group and the non-random nature of the response is unlikely due to methodological variability.

**Fig. 4:**
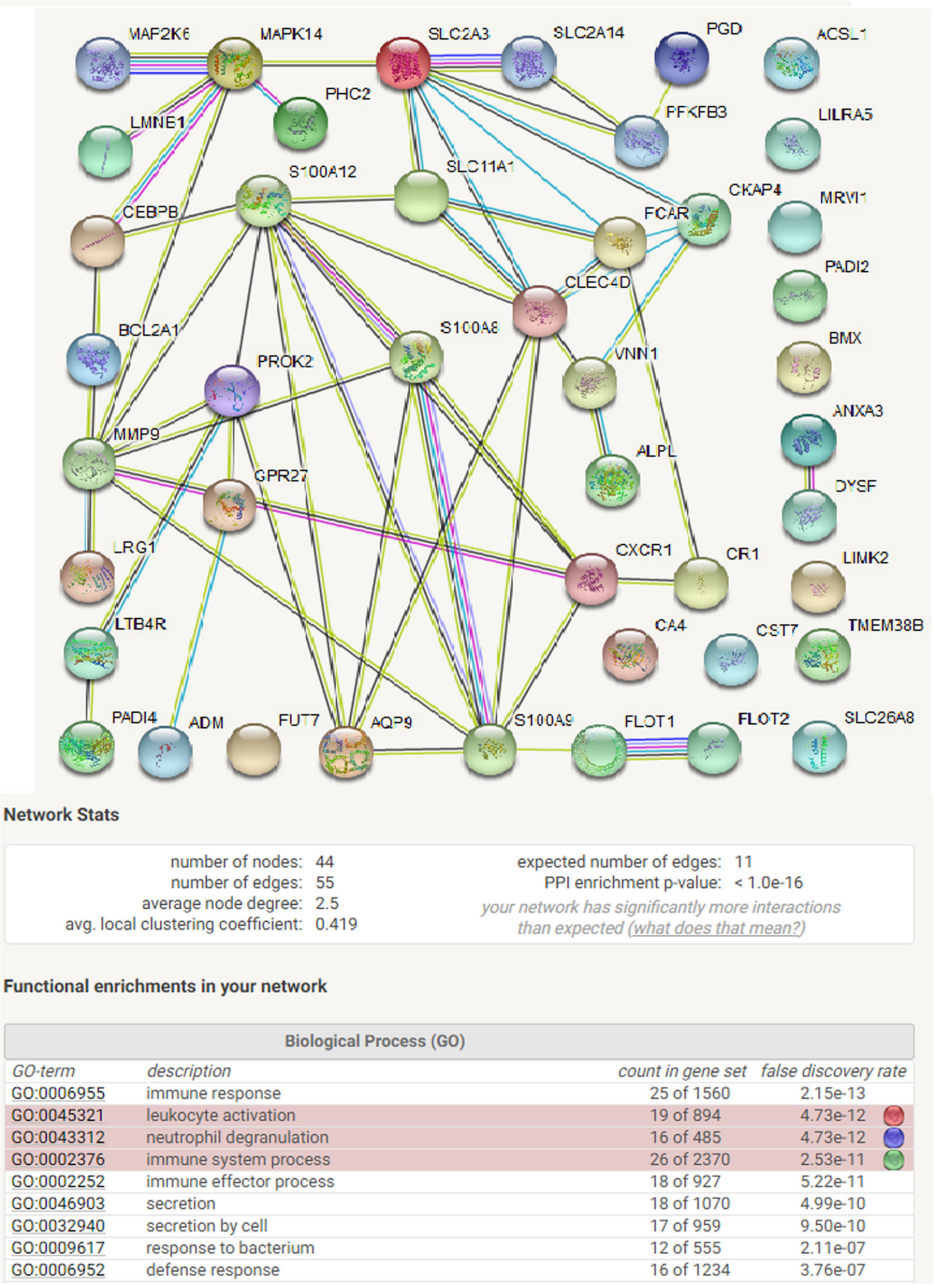
STRING database analysis of 44 genes found to be negatively correlated (*r* ≤ − 0.52465) with the TMEM38B gene. This regulatory cluster is associated with 145 GO pathways that are primarily involved with immune responses such as leukocyte activation (blue, GO:0045321), neutrophil degranulation (red, GO:0043312) and immune system process (green, GO:0002376)

In addition to the 14 positively correlated genes, there were also several gene clusters in which more than 30 genes were identified with negative correlations (r ≤ −.52465; TMEM38B, 43 genes; MMP9, 39 genes and CLEC4D,36 genes). The list of 43 genes associated with TMEM38B were evaluated with the STRING-db to determine if any of these genes shared pathway relationships and the results are depicted in Fig. 4. These 44 genes form associations with 145 different Biological GO pathways with PPI enrichment < 1.0 E-16 and they appear to be primarily involved in mediating immune responses (GO:0006955).

### Localization of highly correlated gene groupings in specific individuals is used to construct a scoring function

The highly correlated cluster of genes identified in Fig. 2, and their coordinated expression responses within certain individuals as depicted in Fig. 3, suggested a second avenue for analysis. The rationale was based on the premise that the coordinated gene activity within a biological pathway would involve multiple genes and this should result in a higher rank-order position for the genes in the activated pathway as well as an increase in the relative number of positionally ranked genes representing that pathway. To explore this possibility, a “Scoring Function” depicting the gene rank position listing was determined for every gene and this analysis is described in Additional file 2, sheet 7 and file 6. Table 1 provides an abbreviated summary of the results. Based on STRING-db analysis, six individuals were identified with gene clusters representing multiple immune pathways with False Discovery Rates (FDR) ≤ E-15. Range /Q3 and kurtosis calculations identified individuals 4, 6, 9, 10, 12 and 33 with multiple immune pathways at FDR’s ≤ E-15 to E-27 (Fig. 3, Table 1 and Additional file 6). The analysis of the 35 control samples identified 6 individuals or 17% of the sample group with genes displaying marked “tailedness”. Moreover, the genes identified in these individuals are involved in the regulation immune function pathways, such as defense response to virus (GO: 0051607) which was identified in 4 of the 6 individuals (11%). A Venn Plot of the genes identified in all three data sets (e.g. data set 1; samples 6, 9, 10, 12 data set 2; sample 7 and data set 3; samples 3 and 4) identified 10 genes common to all three data sets (e.g. HERC5, OAS3, RSAD2, OAS1, MX1, IFI6, IFI44L, IFIT1, OASL and IFIT3). Eight of these 10 genes were previously identified in Fig. 2 with FDR’s ranging from E-15 to E-27 (see Additional files 6, 8 and 9).

**Table 1:**
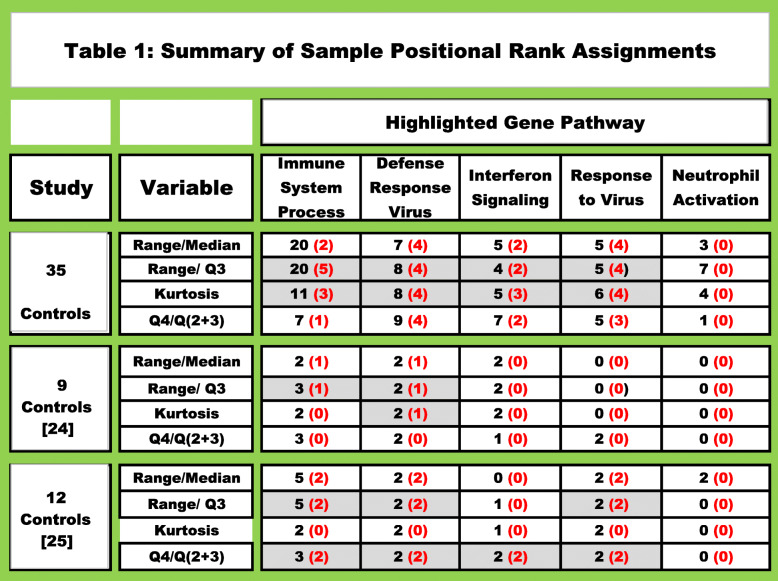
Summary of Sample Positional Rank Assignments

Protein coding gene counts in three data sets were Minimum Value Adjusted (MVA) and rank ordered. The individual with the highest read count for any given gene was assigned a positional rank of 1, the second highest count was assigned a rank of 2, etc. until all of the samples received a positional gene assignment ranking (see Additional file 6). A scoring function was employed to identified a minimum of 1000 positionally ranked genes for each individual. The positionally ranked genes were evaluated to determine if any genes with range/median, range/Q3, kurtosis and Q4/Q(2 + 3) slope values were within a group of the top 300 genes previously identified for each of the selection parameters. For example, in a list of 1000 positionally ranked genes, only genes with a range/median value ≥ to the computed value of the 300th gene would be identified. The genes identified with these four parameters were subsequently evaluated with the STRING db to determine if they were associated with known biological pathways. The black font represents the number of times the designated pathway was ranked in the top 10 pathways in the 35 samples. The number highlighted in red font represents the number of individuals with a False Discover Rate (FDR) < E-15. Samples 4, 6, 9, 10, 12 and 33 all contained one or more pathways with FDR < E-15 to E-42 (Additional files 6, 8, 9). Range/Q3 and range/median calculations were the most robust parameters and identifying the largest number of pathway genes with the smallest FDR. Immune function pathways relating to defense response to virus, response to virus and type I interferon signaling pathways were highlighted in individuals 6, 9,10 and 12 with FDR’s from E-15 to E-26 when range/Q3, range/median or kurtosis values were used for gene identification and STRING-db analysis

### Individuals responding to viruses and pronounced inflammatory responses resulting in elevated numbers of white blood cells contribute to biological variability

Our analysis highlighted sample 33 with neutrophil and leukocyte activation pathways (Additional file 6) and we speculated whether WBC number might be influencing these responses [26, 27]. To address this question, we plotted the WBC differential cell counts for the 35 individuals in our control sample and the results are presented in Additional file 7. Sample 33 clearly contained the largest number of WBC’s and neutrophils. When the cell counts were rank-order, samples 33, 6 and 8 contained a proportionally larger number of WBC’s and neutrophils than the other 32 samples and the removal of those three individuals markedly improved the WBC variation explained by the regression line (WBC R^2^ improves from 0.8492 to 0.9824). This analysis demonstrates that a disproportionate number of WBC’s can also impact gene pathway analysis when the cell numbers are elevated above 10 million WBC’s / ml of blood. The survey of gene trendlines such as IFIT3 based on range/Q3, range/median and kurtosis (Table 1) provided a strategy to identify pathways in which groups of genes appeared to display coordinated expression patterns. The highly correlated group of genes identified in Fig. 2 form multiple protein interactions involving an assortment of different biological pathways as previously illustrated Additional files 4 and 6. The genes identified in Fig. 2 represent virus-induced interferon-stimulated genes [27–30]. When the individuals identified as having coordinated immune responses were removed from the analysis (e.g. individuals 6, 9, 10, 12 and 33, Additional file 6 and Table 1) and the correlations were re-evaluated in the remaining 30 samples, the genes previously correlated with IFIT1 (e.g. r ≥ 0.9579) were reduced from 14 genes to 0. Furthermore, the R^2^ values for the genes identified in Fig. 2 ranged from 0.295 to 0.426 in the sample of 35 individuals were significantly improved to 0.931 to 0.965 after removing the 5 highlighted individuals from the analysis.

### Application of positional rank analysis to survey trendline patterns in control archived RNA-Seq data sets

We retrieved archived Control peripheral blood RNA sequencing files from the GEO database [24, 25, 31] (GSE109313 and GSE112057), processed the raw counts through our pipeline and evaluated genes with mean TPM counts ≥0.5 in the two control data sets (Additional file 11). The resulting lists of genes were filtered to remove nonprotein coding genes and the most robust trendline selection measures including range/median, range/Q3, kurtosis and Q4/(Q2 + Q3) slope values were used to identify genes with the highest positional rank assignments as previously outlined (Additional file 5). A detailed summary of the results is presented in (Additional files 6, 8 and 9).

Since we previously identified individuals in our 35-member Control group that were likely responding to virus-mediated immune challenges (Table 1 and file 6), we evaluated two additional archived Control data sets to determine if the smaller sample groups also contained similar individuals. Employing the 4 parameters used in our previous study (Table 1), a list of positionally ranked genes was assembled with each of the screening parameters. The assembled positionally ranked gene lists were evaluated with the STRING-db [13] to determine if any Biological GO pathways were specifically highlighted in these individuals. STRING-db analysis of the positionally ranked genes identified one individual in Control group b (sample 7, Fig. 3), and 2 individuals in Control Group c (samples 3 and 4, Fig. 3) in which the defense response to virus pathway was significantly elevated (see Table 1 and Additional files 8 and 9 for a detailed summary of the pathway results).

The combined data summarized in Table 1, files 6, 8 and 9 and Fig. 3 demonstrate that positional rank analysis identified from 11 to 17% of the individuals in the three control data sets with gene associations representing virus activated immune pathways. We selected a relatively high benchmark with pathway FDR’s < E-15; but if PPI values < 1.0 E-16 were used for evaluating the gene listings, ~ 20% of the surveyed samples contained individuals in which viral induced immune pathways were identified. The individuals identified as undergoing viral-induced immune responses significantly impact gene expression levels in the pathways that were identified by our analysis, thereby increasing the biological variability of the control sample groups. In addition, the noted increases in the number of WBC’s in some of these individuals were also identified as another source of biological variability in our data set (Additional file 7).

### Can the detected biological responses be explained by technical variability?

**Fig. 5:**
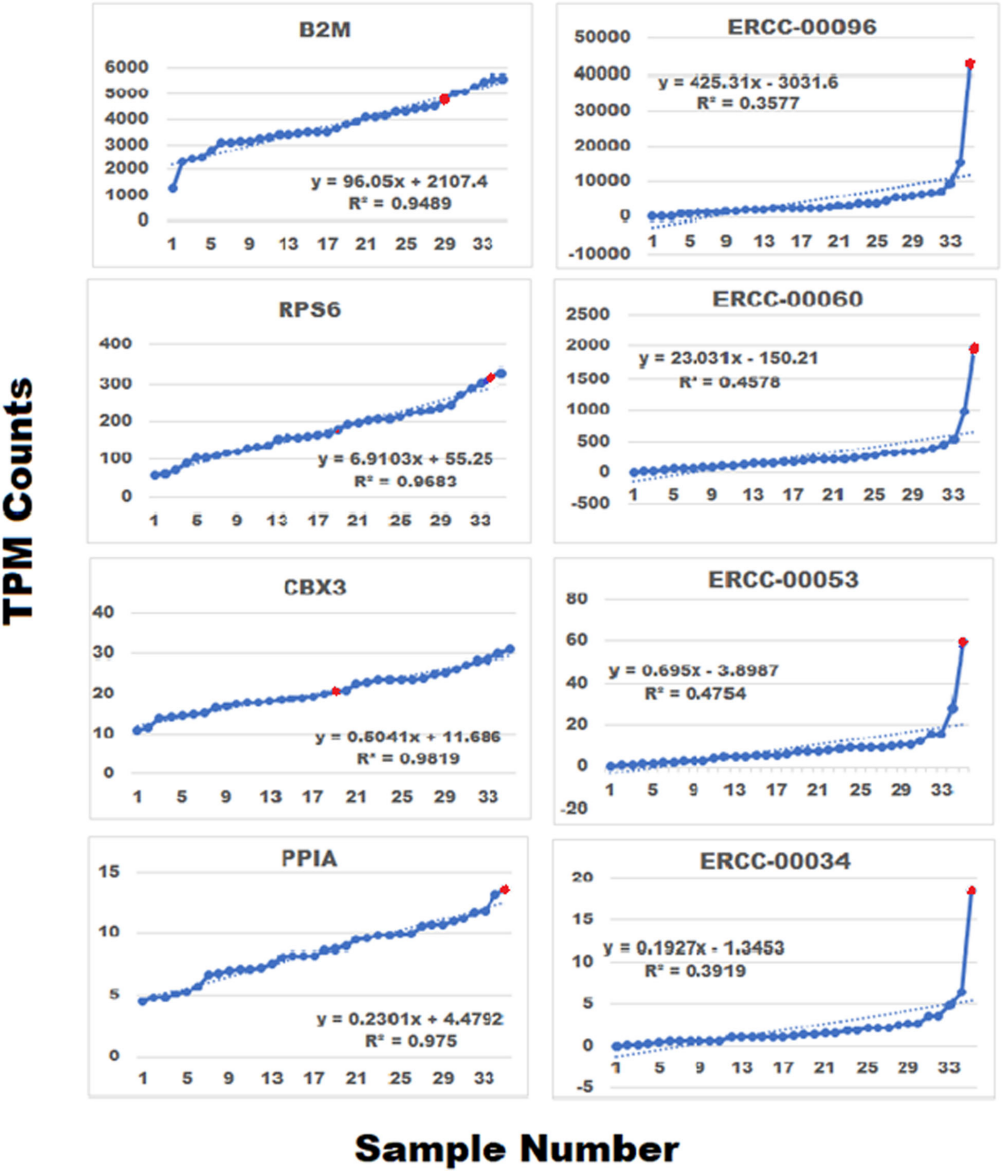
Trendline gene expression profiles remain consistent over a wide range of RNA-seq expression levels. Four genes displaying unadjusted gene counts ranging from 9 to 3836 counts were rank-ordered (left panel). Gene trendline linearity was independent of the level of gene expression. ERCC spike-in standards also showed linearity but the ERCC standard in index 35 (sample 7) was markedly elevated (right panel). However, sample 7 did not display similar deviations in any of the four genes depicted in the left panel of Fig. 5, which suggest a spike-in issue with little or no technical impact on gene counts. Red circles identify sample 7

It is always important to consider how the application of an analytical strategy may impact the results and conclusions of any study. In three separate RNA-seq studies, we noted that 65–70% of the genes follow linear trendline profiles with R^2^ values ≥0.9. We selected 4 genes with unadjusted mean counts ranging from 9 to 3836 to determine if consistent trendline linearity extended across a broad range of gene expression values. The trendline expression profiles for these genes and 4 ERCC standards with similar expression levels are presented in Fig. 5. Similar linear trendlines were observed for both the selected genes and the ERCC standards with the exception of sample 35, representing sample 7, (rank-ordered samples are not listed in numeric sequence, 1–35), in which the ERCC values were consistently larger. The marked deviation from the computed trendline observed in ERCC sample 35 is not reflected in the 4 selected genes in the left panel of Fig. 5, which suggests a potential spike-in issue with little or no technical impact on gene counts.

**Fig. 6:**
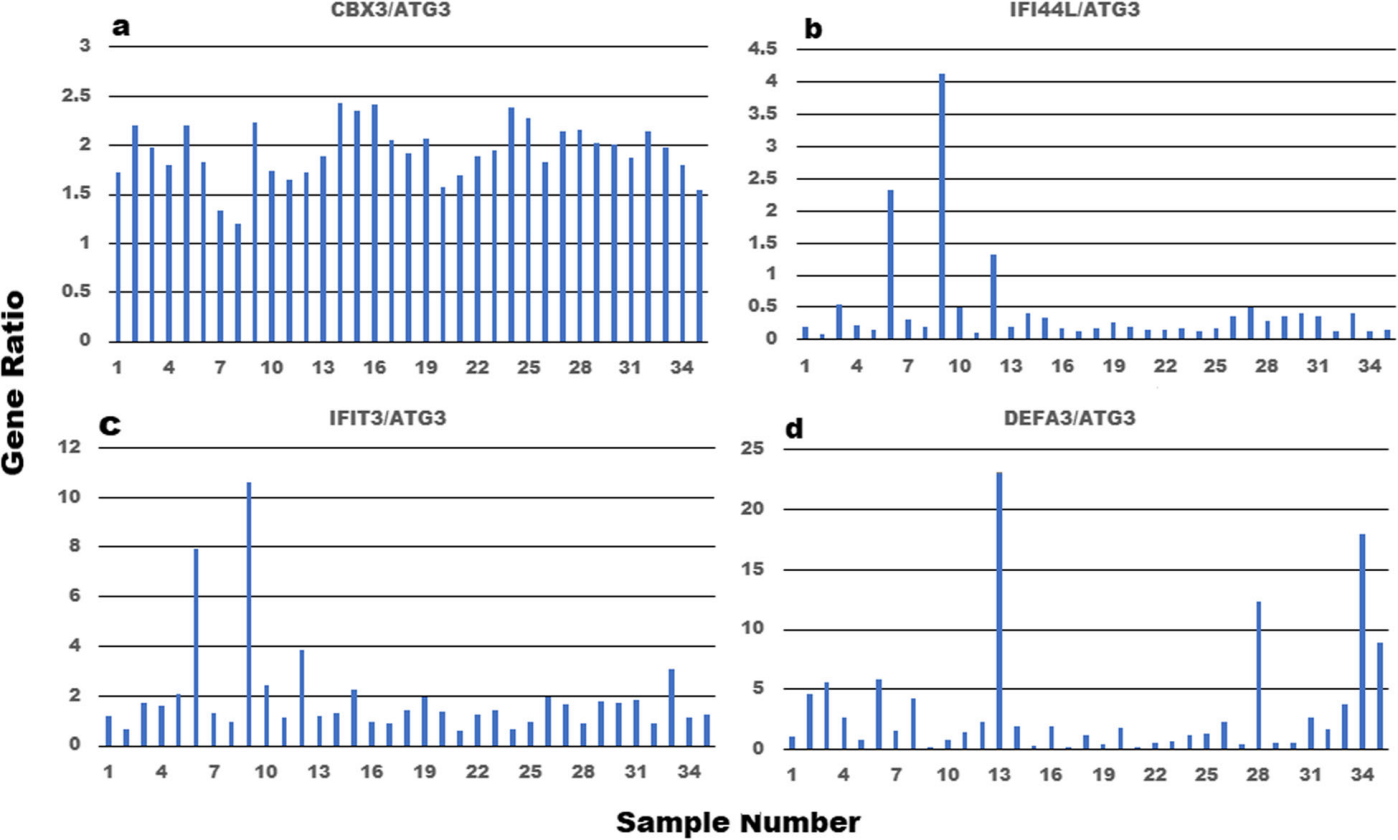
Gene count ratio estimates identify the specific individuals previously identified by String-db analysis. Stable genes identified in three data sets were used to normalize gene expression (see Additional file 10). In panel **a**, the CBX3/ATG3 gene ratios of two highly stable genes are plotted for each of the 35 samples in our study. A 2-fold range of variation is noted between samples 8 and 14. In contrast, when two of the interferon regulated genes were normalized in relation to the ATG3 gene (panels **b** and **c**) samples 9, 6 and 12 were highlighted with ratios 2 to 5-fold higher than noted in panel **a**. The relative response profile of samples 9, 6 and 12 in panels **b** and **c** correspond to the genes correlated with IFIT3 gene previously identified in Fig. 3. In contrast to the IFIT3 and IFI44L response profiles, when the DEFA3 gene was normalized in relation to ATG3, samples 13, 34, 28 and 35 were highlighted. When evaluated in relation to highly stable genes within the data set, the nonlinear gene trendlines identified in our analysis highlight meaningful inter-individual changes in gene expression that cannot be explained on the basis of technical variation

To further evaluate technical and biological variability, we normalized the CBX3, IFIT3, IFI44L and DEFA3 gene expression in relation to the stable ATG3 gene (see Additional file 10). This normalization was performed by calculating gene expression ratios for each of the 35 samples and evaluating the degree of count dispersion across samples when the counts are expressed in relation to a known stable gene. In panel a of Fig. 6, the CBX3/ATG3 gene ratios for two stable genes are presented. The gene ratios for these two genes range from 1.2 in sample 8 to 2.4 in sample 14 (2X range). When the highly correlated and variable expression profiles of the IFIT3 and IFI44L genes, previously identified in Figs. 2 and 3, were evaluated in relation to the ATG3 gene (Fig. 6, panels b and c) the sample gene ratios range from 0.59 in sample 21 to 10.62 in sample 9 (17.9 X range) for IFIT3 and from 0.08 in sample 2 to 4.1 in sample 9 (51 X range). It is also important to note that samples 9, 6 and 12 displayed the largest relative expression levels for both genes as we previously reported in Fig. 3a. Although the highly variable DEFA3 gene displaying an R^2^ value of 0.3956 was not identified among the genes highlighted in Figs. 2 and 3, it also displayed DEFA3/ATG3 gene ratios that varied from 0.2 in sample 9 to 23.1 in sample 13 (116X range). The DEFA3 defensin gene belongs to a family of microbiocidal/cytotoxic peptides found in neutrophile granules that are thought to be involved in host defense responses. The gene expression ratios for the IFIT3 and IFI44L genes identified in samples 9, 6 and 12 (Fig. 6, panels b and c) were substantially greater than the range of values identified among the 35 CBX3/ATG3 gene ratios presented in panel a of Fig. 6. Presumably, the heightened gene ratio responses reflect increased variability resulting from the coordinated biological responses impacting gene expression in samples 9, 6 and 12. In contrast, although the DEFA3/ATG3 gene expression ratios also displayed marked variability, the largest gene ratios were observed in different individuals (samples 13, 28, 34 and 35) suggesting that the biological variability contributing to these changes were different to those impacting samples 9, 6 and 12. Representative sample gene ratio profiles of other stable genes as well as the various genes identified in Fig. 2 were identical to those depicted in Fig. 6, in panels a, b and c. This analysis demonstrates that the relative magnitude of the gene ratio responses identified in samples 9, 6 and 12 were much larger than the 2-fold range of sample-to-sample variation observed for 65–70% of the sequenced genes as depicted in panel a of Fig. 6, and they are unlikely due to technical variability.

## Discussion

RNA-sequencing technology has the potential to contribute significantly to our ability to diagnose and treat many diseases. As clinical medicine relies more heavily on this technology for the application of personalized treatment strategies, it becomes increasingly important to identify and disentangle sources of technological error while preserving and identifying intrinsic sources of biological variability. As previously noted, differential expression tools are impacted by a variety of factors and they are not universally robust to the presence of outlier results from divergent expression data [3, 4, 32–36]. While tools such as EBSeq [33], LFCseq [35], leave-one-out methods [34], median-based approaches, and other software [36, 37], may detect and manage outliers based on the software’s defined criteria, they operate primarily as a “black box” during the differential expression analysis step. These tools may or may not report the outlier, and to our knowledge, they do not provide an intra-group outlier explanation that is readily available to the investigator. Moreover, any outlier detection by current software during DGE is performed at the gene level only. Our approach is focused on providing complementary information prior to DGE. We have evaluated the possibility of creating a methodology that uses existing packages such as Deseq2 [37] or Cuffdiff [36] for intra-group analysis (for example, by using variable-leave-k-out comparisons, where k varies over sample subsets). In our analysis, such an approach was computationally very costly (exploring the set of subsets of 35 samples), and not as descriptively informative. Moreover, our approach extends beyond the gene-level view and reports on the potential impact of divergent values at the network level.

RNA sequencing counts routinely display large differences in their relative gene expression levels, which scales with the mean of the sequenced counts. In previous reports, we developed strategies for stabilizing RNA in whole blood samples and significantly improving RNA recovery during extraction [10, 11]. Here, we used ERCC spike-in standard concentration ratios to minimize amplification and dilutional errors across samples [23], noting that ERCC spike-in mixes were not used in the archived data sets. We also addressed heteroscedasticity by using MVA scaling. This method of scaling is suitable for variability analysis because individual sample-to-sample gene expression levels are adjusted to a common starting point across samples while maintaining the incremental trendline fluctuations of individual genes [23]. In our Control sample, MVA of the 8746 genes reduced the mean and standard deviation by an average of 3.9-fold; however, in highly expressed genes such as B2M the mean and variance are scaled down by as much as 1316-fold (Additional file 2, sheet 3). Nevertheless, the important incremental sample-to-sample gene expression changes were maintained after MVA scaling as illustrated by the gene profiles depicted in panels b and c  of Fig. 1. After MVA, these incremental changes form the basis for identifying the trendline expression profiles (Additional file 4, Panel A vs B). We note that while MVA-scaled data is suitable for trendline analysis, it is important to follow the correct scaling protocol for differential expression analysis by following the specific guidelines of the software that is being employed.

After performing MVA scaling on our data set, we determine that ~ 70% of the 8746 genes in our sample group displayed trendline linearity as assessed by R^2^ values ≥0.9. The application MVA in conjunction with rank-order trendline analysis illustrated that gene expression in this group of genes is consistent with the profile obtained from a normally distributed sample. Moreover, the remaining 30% of the genes that deviate from this linear profile were easily identified and evaluated due their increased variability and dispersion. Our analysis demonstrated that a subset on individuals with tailed gene trendlines in quartile 4, similar to IFIT3 (Fig. 1c), contribute significantly to the variability in the control data set (see Additional file 1). The approach employed in our manuscript is designed to provide an explanatory (and visually inspectable) methodology that can augment existing tools and guide the decisions of the investigator. The statistics used are based on the quantile function [38] and higher moments of distributions (skew) which are readily available in a number of standard statistical software packages. A dramatic example of the variability associated with these genes is depicted by the marked increase in their computed coefficient of variability (Additional file 1, Fig. 4).

By rank-ordering the incremental change in gene expression across samples, we created a unique snapshot or “trendline”. Statistical evaluation of the trendlines identified several robust measures that provided the greatest ability to characterize the biological variability or “tailedness” of the expression values. STRING-db analysis of the genes exhibiting the most pronounced “tailedness” expression profile revealed that these genes were associated with important regulatory networks (Additional file 4). We also demonstrated that positional rank analysis could be used to further evaluate RNA-seq data and identify gene expression variability within specific individuals in the group (see Additional files 6, 8 and 9). The ability to identify and characterize gene trendline properties provides a new and powerful strategy to detect pathway-affiliated genes, and quantify the significance of their associated biological responses via the computed False Discovery Rate in any tissue or sample. To our knowledge, this is the first study in which variability that diverges from standard technical variability has been identified (e.g. The number of Observed Genes per Pathway are identified and statistically quantified by the calculation of False Discovery Rates).

Trendline slope analysis of our 35 control samples identified individuals with gene expression rates in quartiles 1 and 4 that were more than 6-fold greater than the computed slope in quartiles 2 + 3 (32 and 351 genes identified in Q1 and Q4, respectively). Although higher or lower rates of relative gene expression for any given gene in the sample set will contribute to increased variability, rank-order quartile analysis was useful in further characterizing this variation (Fig. 1, panels b and c). One explanation for this response profile is that certain critical regulatory genes were governed by positive or negative regulatory signals [39].

The expression profile of genes such as IFIT3 (Fig. 1b) are difficult to explain on the basis of sample-to-sample variability. The “tailing” trendline observed for the IFIT3 gene indicates that gene expression in about 25% of the individuals was markedly different from the other members of the sample group. These gene trendlines displayed significant non-uniformity (high variability) as illustrated by range/median, range/Q3, skewness and kurtosis measures. Although, kurtosis and skewness calculations identified the degree of “tailedness” of the gene sample distribution, quartile slope analysis provided a more direct measure of these changes. Calculating the slope ratios of Q1/(Q2 + Q3) or Q4/(Q2 + Q3) identified individuals that deviated from the central core of the sample group. STRING-db analysis of the genes displaying these non-linear trendline profiles identified highly integrated pathway associations, as depicted in Figs. 2, 3 and 4, that are involved in the detection and response to a virus. We note that the robustness of the slope ratio calculations is dependent on the size of the sample group (e.g. *n* ≥ 16, Additional file 3).

The characterized gene trendline patterns provided a strategy for evaluating gene associations displaying similar trendline profiles [12]. In our sample group, range/median, range/Q3 and kurtosis calculations were also used to identify gene trendlines displaying marked dispersion and “nonlinearity”. Gene clusters identified with these parameters were subsequently evaluated with the STRING-db and the results were summarized in Table 1. STRING-db analysis as well as correlation studies demonstrated that the genes displaying pronounced non-linear trendline properties resembling the IFIT3 gene, play a prominent role in the mobilization and activation of specific immune pathways (Additional files 4, 6, 8 and 9 and Figs. 2, 3 and 6). We identified specific individuals in the control data sets expressing a disproportionate number of genes in the defense response to virus pathway (GO:0051607) with Observed Gene Count pathway assignments ranging from 4 to 29 genes and FDR’s from E-2 to E-26 in a pathway containing only 181 genes. Our results are suggestive of intra-group differences beyond technical variability. Under the assumption that “methodological variability” is mostly stochastic, the emergence of highly correlated regulatory pathways (Fig. 2) with significant FDR’s identified during STRING-db analysis (Additional file 6) would be highly unlikely. This observation was further supported by noting that when the interferon-mediated genes identified in Fig. 2 were expressed in relation to highly stable genes such as ATG3 (Fig. 6), samples 9, 6 and 12 were consistently identified with the largest gene ratios. Moreover, random selections of 300 genes from the entire list of 8746 genes never resulted in FDR’s ≤ E-6 (*n* = 5, Additional file 3). While we cannot exclude the possibility that technological and methodological error may influence our findings, the results presented in Table 1 and in Figs. 5 and 6 support the conclusion that technical and methodological error had nominal impact on our findings.

Intra-group identification of 11–20% of the individuals in three separate data sets as responding to a viral-induced immune response is an important observation that should be considered prior to differential expression analysis. In addition, other related pathways involving defense response (GO:0006952), response to virus (GO:0009615) and the type 1 interferon signaling (GO:0009615) pathways were also routinely found among the top 10 identified pathways in conjunction with the defense response to virus pathway. Identification of individuals that exhibit a defense-related response within an experimental group is consistent with the time-dependent activation and transition of the immune system from the detection of a foreign object to a defined immune response [27–30].

We have noted that changes in gene expression may represent 5 to 50-fold deviations from the median expression level as illustrated for the IFIT3 gene in panel c of Fig. 1. Current analytical pipelines for DGE are designed to remove “outliers” which display more than 30% disagreement [1]. Based on our results, these protocols remove the gene counts of individuals displaying the greatest biological variability thereby diminishing the opportunity to detect cases of interest wherein a contributing physiological basis for variability may be at work. The removal of specific individuals that appear to be responding to a viral challenge may remove a previously unrecognized source of biological variation. While our approach informs the investigator about these cases, it is beyond the scope of this report to determine how the mitigation of this source of biological variability will influence DGE during principal component analysis.

Identifying and characterizing the genes assigned to the highest positional ranks enabled us to identify individuals with an increased number of genes in certain GO pathways in our control group (Additional file 6) as well as in the archived data sets (files 8, and 9). Our analysis identified asymptomatic individuals who may have been responding to an immune challenge; for example, a recent infection, an immunization, or a response to one of the many latent viruses we commonly harbor in our bodies [40]. A broad array of genes in alternative gene expression pathways were also identified in our analysis but they did not reach a statistical level of significance (see Additional files 6, 8 and 9). These genes also contribute to the biological variability in the various sample groups but they do not appear to introduce an inordinate degree of variability. Although the impact of differences in WBC number and the detection of interferon-regulated genes involved in inflammatory response have been previously described [26–30], we believe this is the first report in which these responses have been quantified and localized to specific individuals within control data sets.

After MVA and establishing trendlines, several calculations including estimates of R^2^ were prepared. R^2^, a measure of the variance explained by regression, provided an estimate of the linearity of every gene in the data set. We have ranked the R^2^ values for the three data sets in descending order and identified 15 genes with R^2^ values > 0.96 that had raw mean counts > 5 and that were common to all three data sets (Additional file 10). Within this group of genes, MVA reduced the raw means by 12-fold and identified genes with very stable trendlines that may be useful as internal reference gene candidates for evaluating RNA sequencing results (e.g. Fig. 6) or as qRT-PCR gene markers as previously suggested by Stamova et al. [41].

The application MVA in conjunction with rank-order trendline analysis provides a strategy for identifying previously unrecognized sources of biological variation. However, it should be noted that the expression levels of ERCC spike-in standards, as depicted by sample 7 in Fig. 5, may also be impacted by the initial composition of the RNA sample [3, 19]. Our approach is helpful towards tackling the difficult question of how variability may impact the conclusions of previous studies. This analysis is simple to perform and requires a minimal amount of computational time. Therefore, it may significantly contribute to the clarity of our understanding of the manner in which previously unrecognized sources of biological variability may have biased or confounded the experimental analysis with minimal overhead. Although differential gene expression analysis remains an important application in RNA-seq studies, the detection of regulatory pathways containing genes that may not otherwise be identified as differentially expressed provides new insight into the complex mechanisms governing human diseases.

## Conclusions

Rank-order analysis of the MVA gene expression values in conjunction with R^2^ calculations revealed that 65–70% of the sequenced genes display a linear “baseline” level of gene expression across the data set. Statistical measures, such as range/median, range/ Q3 and kurtosis, that characterize the “tailing” properties of some gene trendlines, were used in conjunction with databases such as STRING-db or PANTHER-db to identify and quantify gene pathways contributing to biological variability within and across three different sample groups. Pathways relating to viral-induced immunological responses were identified in 11–20% of the 54 individuals evaluated in our combined studies. This previously unrecognized source of variability may confound or obscure DGE results and mask important conclusions obtained from immunological studies.

Minimum Value Adjustment (MVA) scaling significantly reduced the average mean and standard deviation in the data set by 3.9-fold among the 8746 protein-coding sequenced genes. However, MVA preserved the unique incremental sample-to-sample changes in gene expression across individuals following rank-ordering. Furthermore, the resulting range and magnitude of the incremental changes in gene expression following MVA were markedly similar even though the RNA was extracted and processed differently in the three data sets evaluated in our study. While MVA may also reduce some of the variability that is commonly introduced when data sets are sequenced in different laboratories and at different times, its designated utility in our studies is in the analysis of intra-group comparisons.

## Methods

### Sample collection, RNA extraction, sequencing and data analysis

Blood samples were collected from 35 healthy adults according to a protocol approved by Chesapeake Research Review, LLC (#Pro:00009509). Blood samples were used for complete blood cell analysis and RNA extraction [10, 11]. RNA was DNase-treated and submitted to the University of Cincinnati Genomics, Epigenetics and Sequencing Core Facility for RNA sequencing. The computational pipeline employed in this study has been previously described [23].

The RNA extracted from the 35 control samples passed quality control assessment prior to the addition of External RNA Controls Consortium ExFold RNA spike-in mixes (ERCC: Ambion, 4,456,739; Foster City CA) and removal of ribosomal and globin RNA from the samples (Illumina GZG1206; San Diego, CA). The cDNA libraries were processed according to standardized Illumina protocols and sequenced on the Illumina HiSeq 2000 platform. Fastq data files containing 53–77 million single-end reads were trimmed and processed for data analysis. Reads were aligned to reference genome GRCh37.p13[hg19] using the BowTie2 aligner supporting gapped alignments as previously described [23, 36].

### Processing RNA sequencing data files

Data files containing 10–13 thousand transcripts were imported into an Excel spreadsheet and matched to a list of human protein coding genes identified at the Gene Ontology website [20–22]. To optimize our analysis in databases such as STRING [13–15] and PATHER [42, 43], we removed noncoding genes (filter) from further consideration [12]. When noncoding genes were removed, False Discovery Rate (FDR) estimates were improved during STRING-db pathway analysis thereby increasing the sensitivity of gene pathway identification. Transcripts that were not identified as protein-coding genes were assigned to a separate “Discard” data sheet. After identifying the protein coding genes, samples with gene counts < 0.5 were replaced with 0 and genes with means < 0.5 counts were eliminated from further analysis [12].

Single genes that mapped to multiple genomic locations (Copy Number Variants, CNV [44]) were identified and read counts were combined under a single identification convention (e.g. The Control data set contained 66 CNV genes with duplicate/triplicate gene location listings).

The final raw data processing step was the Minimum Value Adjustment scaling procedure described below (MVA, Additional files 1 and 3). More detained information relating to the data entry process is provided in Additional file 2.

### Data processing, normalization strategies and statistical analysis

In our control data set, an iterative correction of the length-adjusted ERCC spike-in concentration ratios was used to proportionally adjust for sample processing effects, pipetting errors, dilutional differences and other sources of methodological variability while the archived data sets that did not contain ERCC spike-ins were limited to size factor normalization using the median-to-ratio method as previously outlined [23]. Data were adjusted for sequencing depth and normalized using the Deseq2 median of ratios method [36] before the reads (in units of transcript per kilobase million or TPM) were used in trendline analysis. Our objective was to minimize the impact of methodological variability before applying trendline analysis.

Read counts for various genes ranged over a 5 Log_10_ scale thereby creating large differences in the variance within the sequenced data set. To address the heteroscedastic nature of the raw data, we applied Minimum Value Adjustment (MVA) scaling normalization strategy to our counts. The smallest count for each gene was identified and used to proportionally adjust the remaining samples. MVA = Gene A (Sample Counts _(1 to n)_ / Gene A Minimum Value Count). This adjustment assigns a value of 1 to the smallest count value for every gene. After MVA, all of the incremental changes for the 8746 protein-coding genes across the 35-sample data set fall within a numeric range of 1–60 relative counts. This adjustment of every gene to its lowest common denominator eliminated large comparative differences in the relative magnitude of the observed counts between genes within and across individuals while maintaining the important incremental changes when the adjusted counts are rank-ordered. In addition, the trendline starting point is identical for every gene in the analysis. The removal of inordinately low (counts < 0.5) and nonsignificant gene counts minimized the possibility of inflating the magnitude of the sample-to-sample incremental changes that can be sensitive to very small outliers during scaling adjustments [9].

Statistical calculations were performed using the resident statistical macros provided with Windows 10 Excel software.

### Trendline identification and Analysis

For statistical analysis, the Microsoft Windows Excel Platform and its statistical and mathematical functions were used. In order to organize and manage the data, software was developed to streamline and augment our analysis [12]. RNA sequencing counts were imported into Excel, duplicate gene entries were identified and removed, gene counts that were below a user defined value were checked for meaningful gene expression levels and nonprotein coding genes were removed (optional) before performing Minimum Value Adjustment scaling (see Additional file 2 for details and examples of data output).

A primary objective of our study was to determine whether rank-order analysis was a useful strategy to identify sources of intra-group biological variability that otherwise remain difficult to detect in RNA-seq data. In order to omit nonprotein coding genes from the sequencing results, each gene was surveyed to determine whether the transcript was a known protein coding gene. We used the Gene Ontology website to provide a list of 19,623 known human protein coding genes [20–22]. Genes that were not recognized as protein coding genes are listed as “filtered units” and they can be further analyzed if desired.

Two markedly different gene trendline expression profiles are presented in supplement 1 to illustrate how linear regression analysis and other calculations can be used to analyze rank-ordered gene count data. Trendlines were constructed for each gene by ranking the adjusted and unadjusted gene counts for the 35 samples in ascending order as described in Additional files 1, 2, 3 and 4. From our list of 35 samples, we identified 15 genes with the largest mean expression values before and after MVA (Additional file 2, sheets 2 & 3). MVA significantly reduced the mean and standard deviation among these genes by 67 to 1316-fold but the Coefficient of Variation was unchanged. Genes were identified in the unadjusted and MVA data sets with R^2^ > 0.9 (linear profile) and R^2^ < 0.9 (nonlinear) trendline patterns. These mathematical calculations were used to further characterize gene trendlines and identify gene groupings (clusters) that shared similar mathematical features. This analysis identified relational associations among genes that would otherwise be indiscernible using more classical analytical procedures. Supplement file 2 describes additional software programing features and examples of data output. An overview of the data input and processing pipeline is summarized in sheet 1 of file 2.

### Gene pathway associations identified by comparing similarities in Trendline characteristics

A variety of statistical calculations were performed on the trendline of each gene. Our results were based on a list of 8746 protein-coding genes with a mean baseline count ≥0.5 counts that were rank-ordered to identify genes with the most prominent trendline property (e.g. range/Q3, kurtosis, R^2^, etc.). Prominent genes considered for additional analysis fell among the top 300 genes characterized by the largest or smallest measurements for any of the computed parameters. For each computed statistical parameter, the identified genes were imported into the STRING database (db) to determine if that parameter linked genes to functionally related protein associations. For example, 300 genes that displayed the largest computed means were identified and imported into the STRING database version 11 [13–15] to determine if the highlighted genes formed known associations within any biological pathway(s). STRING-db analysis was performed using the “high confidence setting of 0.7”. Using various statistical tests, the STRING-db identified the Number of Observed Gene Counts within the original list of 300 submitted genes that were associated with known GO pathways and assigned a False Discovery Rate based on the Observed Gene Count and the total number of known genes in the pathway. Although algorithms employed to compute False Discovery Rates in the STRING-db and Panther-db [13–15, 42, 43] databases are different, False Discovery Rates < E-15 are generally considered significant and the identified Observed Gene Count groupings cannot not be attributed to random gene associations (see file 2 for additional detail).

### Analysis of archived RNA Seq data files

An identical method of analysis was used to evaluate sequencing data obtained from peripheral blood samples extracted and processed with different methodologies in order to determine whether the MVA scaling and rank-ordering methods could identify similar changes in gene pathway affiliations among Control samples in two archived data sets. We downloaded data files from the NCBI Gene Expression Omnibus site [31] containing sequencing data obtained from the peripheral blood of 9 Controls (GSE109313, [24]) in one data set and 12 Controls in a second data set (GSE112057, [25]). Blood samples in the first data set were extracted with the PAXgene RNA blood extraction kit and further processed to prepare poly(A) selected and ribo-depleted RNA-seq libraries. In data set 2, blood was collected via Tempus Tubes and RNA was extracted with the Tempus Spin RNA isolation kit.

## Supplementary Information

**Additional file 1.** Rationale for identifying sources of biological variation in RNA sequencing data.

**Additional file 2.** Summary of Statistical Measures used to Evaluate Gene Expression before and after Minimum Value Adjustment (MVA).

**Additional file 3.** The Identification of Gene Trendlines with “Tailing” Profiles Using Quartile Slope Analysis.

**Additional file 4.** STRING db Pathway Identification Based on the Analysis of the Top 300 Genes Identified by the Designated Statistical Measures.

**Additional file 5.** Examination of Intraindividual Gene Rankings to Identify Individuals Displaying Coordinated Gene Regulatory Activity.

**Additional file 6.** STRING db Analysis of Intra-individual Positional Gene Rankings in 35 Control Samples Based On Range/Median, Range/Q3, Kurtosis and Q4/Q(2 + 3) Slope Calculations.

**Additional file 7.** Rank-Ordered Distribution of White Blood Cells Among 35 Control Samples.

**Additional file 8.** STRING db Analysis of Intra-individual Positional Gene Rankings In 9 Archived Controls Based on Range/Median, Range/Q3, Kurtosis and Q4/Q(2 + 3) Slope Calculations.

**Additional file 9.** STRING db Analysis of Intra-individual Positional Gene Rankings In 12 Archived Control Samples Based On Range/Median, Range/Q3, Kurtosis and Q4/Q(2 + 3) Slope Calculations.

**Additional file 10.** Reference Genes with Raw Counts Greater than 5 and R^2^ Values > 0.9 in three separate RNA sequencing Studies.

**Additional file 11.** Control Reference Data Files.

## Data Availability

Copies of the three processed unadjusted data files that were used in this study are provided in Additional file 11. Sheet 1 contains our control data files and sheets 2 and 3 contain the Control data files from the Mangul et.al [24]. and Mo et.at. reports [25] that were processed through our pipeline. The original RNA sequencing data files for the 35 controls described in this study have been archived at the NCBI Gene Expression Omnibus site with accession number GSE169359 (https://www.ncbi.nlm.nih.gov/geo/query/acc.cgi?acc=GSE169359). The additional archived data files referenced in our study are available at NCBI Gene Expression Omnibus site (https://www.ncbi.nlm.nih.gov/geo/) containing sequencing data obtained from the peripheral blood of 9 Controls (https://www.ncbi.nlm.nih.gov/geo/query/acc.cgi?acc=GSE109313) [24] in one data set and 12 Controls in a second data set (https://www.ncbi.nlm.nih.gov/geo/query/acc.cgi?acc=GSE112057) [25].

## Abbreviations

FDR: False Discovery Rate
GO: Gene Ontology
MVA: Minimum Value Adjustments
RNA-seq: RNA sequencing
SD: Standard Deviation
CV: Coefficient of Variation
db: Database
WBC: White blood cells
DGE: Differential gene expression
