# Supplementary material for: Strategies for detecting and identifying biological signals amidst the variation commonly found in RNA sequencing data
Source: BMC Genomics. 2021 May 3;22:322. doi: 10.1186/s12864-021-07563-9 (PMC8091537; doi:10.1186/s12864-021-07563-9)
Supplement: Supplementary file 1 — Additional file 1. Rationale for identifying sources of biological variation in RNA sequencing data. [file 12864_2021_7563_MOESM1_ESM.docx]

**Additional file 1:**

**Rationale for identifying sources of biological variation in RNA sequencing data.**

When RNA sequencing counts are ranked in ascending order, different patterns of gene expression can be identified as illustrated by the expression profiles depicted in Figure 1 of the manuscript. To efficiently screen data files containing thousands of genes, it would be useful to identify selection criterion that could be employed to detect and categorize changes in gene expression within a data set. To demonstrate how various statistical parameters might identify changing patterns of gene expression, software was developed to perform a variety of statistical calculations. To illustrate this point, we have used the Excel Randbetween function to select 30 numbers within an arbitrary range of 16 to 43. The numbers were subsequently ranked in ascending order and they are graphically depicted in Figure 1A. As expected, the computed regression line for the data set depicts a straight line when numbers are randomly selected from a specified range of values (e.g. 16-43). In this example, the computed slope is approximately 1 and the regression line explains a large degree of the variation as evidenced by the computed R^2^ value of 0.9908. An analysis of the regression residual sums of square for sample A (Figure 2), illustrates that the residual error is small and randomly distributed along the regression line with a maximum dispersion of < ± 2 units. Genes that display R^2^ values > 0.9 follow this profile when the counts are rank ordered and evaluated by regression analysis. In three separate studies, 65-70 % of the sequenced genes, in three separate control samples, follow this trendline profile.

However, some genes can display count values that are 10 to 50 times greater than the sample median. To illustrate this point, we removed samples 5,10,15,20 and 25 and replaced those values with numbers that are 5, 10,15, 20 and 25X greater than the computed median (i.e. 29.5) of Sample A. The resulting values were ranked in ascending order as illustrated in Sample B of Figure 1. The rank-ordered plot clearly depicts a bimodal pattern. When comparing the computed results obtained from Samples A and B, the slope was increased from ~ 1 to 13, and the R^2^ value was reduced from ~1 to 0.4. The Coefficient of Variation (CV) increased from 29.4 % in sample A to 183.4 % in sample B and the residual regression sums of square were dramatically increased by 400-fold relative to sample A (SS B 600/ SS A 1.5) as illustrated in Figure 2, panel B. Moreover, the dramatic change in slope produced coordinated changes in the residual error along the regression line that is markedly different from the random fluctuations observed in Sample A. Genes such as IFIT3 depicted in Figure 1 of the manuscript, display similar trendline profiles with a slope > 1 and R^2^ values < 0.9 and they also exhibit dramatic increases the CV and in the residual Sums of Square attributable to regression. Genes displaying large count dispersion based on the linear regression model can display significant increases in the residual sums of square thereby highlighting the fact that the incremental change in the rank-ordered counts have markedly changed and may therefore identify a shift in the level of gene expression. We propose to use rank-order analysis to identify genes displaying disproportionate shifts in sample variance and determine whether these trendline responses are linked to other genes that are known to contribute to biological responses.

Although the median values for the two data sets presented in panels A and B of Figure 1 are similar, 29.5 vs 31.5, the sample range increased from 27 in Sample A to 721.5 in Sample B (26.7-fold). The Coefficient of Variation between Samples A and B increased from 29.5 to 183.4 % (6.2-fold). In a normally distributed sample, such as Sample A, the range is approximately = to the median and the Range/Median Ratio is approximately 1 (e.g. 0.915, sample A). In contrast, in sample B, the Range/Median Ratio is increased to 22.9. Calculations such as Range/Median Ratio, Range/Quartile 3 Ratio, Kurtosis, etc. which can be easily computed and employed as a screening tool, can be used to identify and characterize genes displaying these non-linear trendline profiles. In our analysis we have evaluated a variety of statistical measures, as illustrated in Additional file 2. Values such as mean, standard deviation, range/median and slope computed from the trendlines of specific genes can be sorted in descending order, and the top-ranking genes identified by these parameters can be evaluated with databases such as “STRING” or “PANTHER” to determine if the list of genes identified by the computed parameter is useful in identifying genes with similar expression profiles.


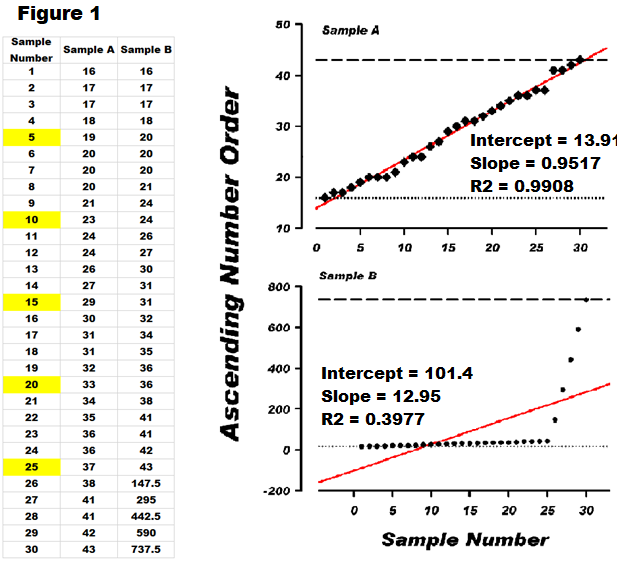


**Figure 1: Trendline profiles depicting a normally distributed (Sample A) and a skewed data set (Sample B) following rank ordering and regression analysis. Samples representing a normally distributed group of data points form a linear profile when the values are ranked in ascending order and sequentially plotted as illustrated in panel A of Figure 1. The median value (29.5) is approximately equivalent to the observed sample range (27). In contrast, a data sets such as Sample B, containing markedly skewed values has a greatly expanded range (721.5) while the median value is only modestly changed (31.5). Therefore, the calculation of the Range/Median ratio for sample A and B produce significantly differ results (Range/Medium A= 0.915 vs 22.9 Sample B). This calculation identifies individuals within the sample group that display marked deviations from the median that may have been previously considered as “outliers” and omitted from subsequent analysis or normalized by mathematical transformations. In order to determine if there might be a physiological basis for these observations, we have used STRING db analysis to determine whether the genes displaying these markedly nonlinear expression profiles are known to interact with other genes displaying similar expression profiles. String db analysis of the genes displaying these nonlinear expression profiles identified gene groupings sharing distinct immune-related biological pathway responses. This coordinated activation of multiple genes within a distinct biological pathway may identify previously unrecognized regulatory events and sources of biological variability within the data set.**

**Figure 2**

**Figure 2: Distribution profile of residual variance in normally distributed and skewed samples. Linear regression residual sums of squares plot of the rank-ordered data depicted in Samples A and B of Figure 1. Rank-ordered incremental changes in gene trendline expression that follow a linear profile display small random fluctuations in residual sums of square while nonlinear samples such as sample B display large positive or negative synchronous changes in the residual sums of square. R^2^ values that are < 0.9 identify genes displaying non-linear incremental expression profiles with marked increases in the residual sums of squares.**

**The Impact of Gene Count Heteroscedasticity on Analysis of Variance Sums of Square**

In addition to the variability in the Sums of Square that is introduced by a change in the level of sample dispersion as depicted by samples A and B, RNA-seq data display large changes in the level of gene-to-gene expression that can vary over a 5 Log_10_ scale. Changes in the mean level of gene expression dramatically impact the variance and contribute significantly to the heteroscedasticity observed in sequencing results. In our control data set, we have looked at this variance by applying One Way Analysis of Variance with repeated measures to a group of 6282 genes containing 35 samples and having TPM counts ≥ 0.5 counts (Table A). In our studies, we have used Minimum Value Adjustment to mitigate the mean and variance dispersion within the data set. This calculation provides a strategy for adjusting each gene to their lowest common denominator, thereby significantly reducing the total variance. The change in the distribution of the Sums of Squares after MVA gene counts is depicted in Table B. It is important to note that after MVA the total variance Sums of Square was reduced by 99.89%. Moreover, the relative proportion of Within and Between groups Sums of Square was significantly shifted (Table A vs B). For example, the Within group Variance was increased from 19 to 61 % of the Total Sums of Square but the computed F-values was unchanged by MVA. This response can also be seen in the mean and standard deviation plots of the unadjusted and MVA data sets in Figure 3. Adjusting gene counts to their lowest common denominator highlights the incremental changes in the rank order of the gene counts and minimizes the variation introduced by the relative magnitude at which the gene was initially expressed. MVA removes the confounding influence that is imparted by the relative magnitude of the sequencing response without altering the incremental relationship of the samples within the data set. It may be important to note that MVA may also remove some of the variability that has been described when sequencing results are processed with different methodology.

Table A: One Way Analysis of Variance of the TPM Counts of 6283 genes

| *Source of Variation* | *SS* | *df* | *MS* | *F* | *P-value* | *F crit* |
| --- | --- | --- | --- | --- | --- | --- |
| Between Groups | 1156821603 | 6282 | 184148.6 | 143.0067 | 0 | 1.029978 |
| Within Groups | 275079403 | 213622 | 1287.692 |  |  |  |
| Total | 1431901006 | 219904 |  |  |  |  |

Table B: One Way Analysis of Variance of MVA TPM Counts of 6283 genes

| *Source of Variation* | *SS* | *df* | *MS* | *F* | *P-value* | *F crit* |
| --- | --- | --- | --- | --- | --- | --- |
| Between Groups | 582169.7 | 6282 | 92.67267 | 21.81719 | 0 | 1.029978 |
| Within Groups | 907400.1 | 213622 | 4.247691 |  |  |  |
| Total | 1489570 | 219904 |  |  |  |  |

**Figure 3**


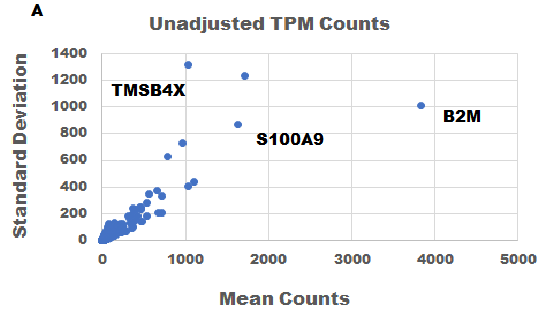


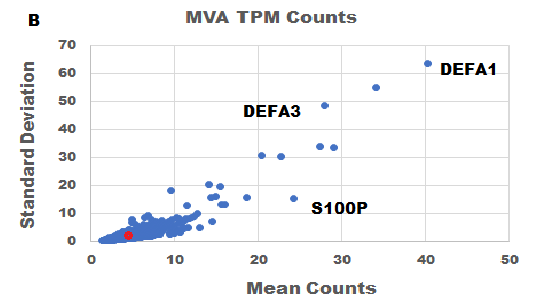


**Figure 3: Minimum Value Adjustment significantly changes the gene groupings based on their computed variability (e.g. Standard Deviation). Plot of mean and standard deviations of 6283 genes before (A) and after (B) Minimum Value Adjustment (MVA). The adjustment of the gene counts to their lowest common denominator by MVA significantly reduce the mean and standard deviation scale (Plot A vs B). The genes that were highlighted in Panel A, B2M, S100A9 and TBSB4X are no longer prominently identified in Panel B. The mean and standard deviation for gene B2M was reduced from 3836.3 ± 1010.3 to 2.91 ± 0.76 in Panel B (red circle). The large reduction in the Total Sums of Square (Table B) improves the ability to identify Within and Between Group changes that may identify significant sources of biological variability.**

**Estimates of sample variance and dispersion based on Coefficient of Variation.**

Although Minimum Value Adjustment significantly reduces the standard deviation it has no impact on the Coefficient of Variation calculation (CV). Therefore, it was of interest to evaluate the trendline profile of the CV values after rank-ordering the data. Figure 4 depicts the CV trendline profile of the 6283 genes evaluated in our control sample. Greater than 90 % of the genes display CV values ranging from 20 to 40 % that are characterized by small gradual linear increases in variance across the data set. Approximately 370 genes have CV values > 55%. The same group of genes that are identified on the basis of Range/Median Ratio, Range/Quartile 3 Ratio, Kurtosis calculations also display markedly increased CV values as depicted in Figure 4. The marked increase in variability of this small group of genes dramatically increases the variability of the data set. We believe this variability contributes to what is generally known as biological variability. Therefore, an analysis of the genes displaying this marked increase in variance may identify sources of biological variation that have not been previously considered.

**Figure 4**

**
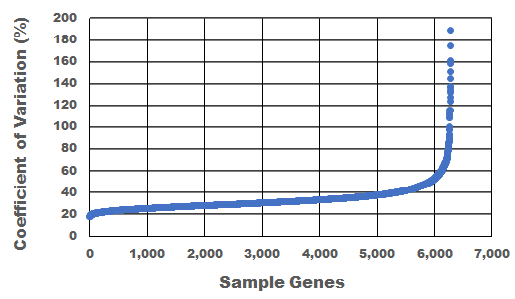
**

**Figure 4: Rank-order distribution of the Coefficient of Variation data highlights marked changes in a small sub set of ~ 300 genes. In contrast to the sample Standard Deviation data, Minimum Value Adjustment does not alter CV values. Therefore, multiple calculations such as Range/Median Ratio, Range/Quartile 3 Ratio, Kurtosis, CV, etc. can all be used to detect genes that display marked increases in sample dispersion and variability.**

**Application of Quartile Analysis to detect changes in gene expression.**

A fundamental principle of linear regression analysis is based on the observation that obtaining a sample from a normally distributed population and plotting the values in ascending order produces a linear numeric array. Therefore, if numbers are randomly generated from a defined range of values (Excel; RANDOMBETWEEN) one can create a linear array when the number sequence is rank-ordered and sequentially plotted as previously depicted in Figure 1, panel A. The approach of sorting and plotting expression counts provides an approximation for obtaining the Quantile Function, which is one of the ways of prescribing a probability distribution. In more formal terms, the quantile function, Q, of a probability distribution is the inverse of its cumulative distribution function. For our statistical application to expression counts, we need to know key percentage points of a gene expression data set in the sample population. For example, we may consider the median and 25% and 75% quartiles in order to assess the statistical significance of an observation whose distribution is known. The approximation of the quantile function provides a straightforward method for reading gene count values from the graph of the function we have obtained by sorting the data and plotting it in increasing order. The formal statistical basis and several applications of quantile functions have been extensively discussed [41]. Rank-ordering and plotting numbers from a normally distributed population yields correlation values r > 0.95 (R^2^ > 0.9025). Therefore, if gene sequencing results are rank-ordered and display a linear expression profile with R^2^ values > 0.90 after ranking, the gene counts may be considered to represent counts derive from a normally distributed sample.
