# Supplementary material for: Strategies for detecting and identifying biological signals amidst the variation commonly found in RNA sequencing data
Source: BMC Genomics. 2021 May 3;22:322. doi: 10.1186/s12864-021-07563-9 (PMC8091537; doi:10.1186/s12864-021-07563-9)
Supplement: Supplementary file 3 — Additional file 3. The Identification of Gene Trendlines with “Tailing” Profiles Using Quartile Slope Analysis. [file 12864_2021_7563_MOESM3_ESM.docx]

**Additional file 3:**

**The Identification of Gene Trendlines with “Tailing” Profiles Using Quartile Slope Analysis.**

RAnGER can be employed to segregate gene trendlines into three categories according to their 1^st^, 2^nd^+3^rd^ and 4^th^ quartile sample groupings. For 35 rank-ordered samples, eight individuals were assigned to quartiles 1 and 4 respectively with the remaining individuals to quartiles 2 and 3. Each line segment was regressed and the slope ratios of Q1/(Q2+Q3) and Q4/(Q2+Q3) were determined. A total of 5735 genes displaying R^2^ values ≥ 0.9 were found to have Q1/(Q2+Q3) or Q4/(Q2+Q3) slope ratios < 6. Based on this observation, genes displaying unique “tailing” characteristics were identified as having Q1 and/or Q4 slope ratios > 6 x the computed slope of the rank-ordered counts for individuals in quartiles 2 and 3 (manuscript, Figure 1B and 1C). After MVA, 34 (0.6 %) genes were identified displayed Q1/(Q2+Q3) ratios ranging from 6.0 to 11.52 and 573 (6.5 %) genes with Q4/(Q2+Q3) ratios ranging from 6.00 to 57.02 (IFI27). The identified genes were subsequently examined with the STRING db.

The 34 genes identified with Q1/(Q2+Q3) ratios ≥ 6 did not share a statistically significant number of protein associations with any Gene Ontology (GO) biological pathway network. However, STRING db analysis determined that 300 of the 573 genes with the largest Q4/(Q2+Q3) ratios ≥ 6 were associated with 47 different biological GO Pathways involving from 4 to 92 of these genes. The top 32 pathways were immune function pathways with False Discovery Rates (FDR) as low as 2.64 E-12 (PPI < 1.0 e-16) (GO.0051607, defense response to virus, 25 of 181 genes). The genes identified with Q4 trendline “tailing” patterns form multiple associations in pathways involved in the integration and coordination of a variety of physiological processes. Therefore, based on STRING-db analysis and the low False Discovery Rate for this cluster of genes, it is unlikely that these “tailing” profiles are due to methodological variability. Furthermore, the selection of 5 sets of 300 genes by random sampling from the original 8746 protein-coding genes in this study did not result in the detection of any pathways with PPI < 1.0 E-3 or with FDR < 8.3 E-5.
