# Supplementary material for: Strategies for detecting and identifying biological signals amidst the variation commonly found in RNA sequencing data
Source: BMC Genomics. 2021 May 3;22:322. doi: 10.1186/s12864-021-07563-9 (PMC8091537; doi:10.1186/s12864-021-07563-9)
Supplement: Supplementary file 5 — Additional file 5. Examination of Intraindividual Gene Rankings to Identify Individuals Displaying Coordinated Gene Regulatory Activity. [file 12864_2021_7563_MOESM5_ESM.docx]

**Additional file 5:**

**Examination of Intraindividual Gene Rankings to Identify Individuals Displaying Coordinated Gene Regulatory Activity.**

We previously determined that range/median, range/Q3, kurtosis and Q4/Q(2+3) slope calculations (Additional file 4) identified genes displaying “tailedness”. Therefore, we used these calculations to determine whether genes displaying this expression profile could be identified within specific individuals. To study this issue, we created a matrix in which MVA gene counts were assigned a positional rank [12]. For example, if individual 9 in Panel A of Figure 3 had the largest expressed counts for the IFI44L gene, it would be assigned a positional rank of 1. If the second largest value for IFI44L was observed in sample 6, it would be assigned a positional ranking of 2 and if the smallest gene count for IFI44L was in sample 28, it would be assigned a positional rank of 35. This positional ranking process was repeated for each gene and a matrix containing the positional rankings of 8746 genes for each of the 35 individuals was constructed. Although the relative magnitude of gene expression varied among individuals, the positional gene rank assignments are individually unique in relation to their relative ranking within the sample group. Our previous analysis focused on the identification of gene pathway associations within the sample while this analysis identifies gene pathway associations within specific individuals in the sample.

A total of 1000 genes with the top positional rankings were extracted for each of the 35 controls and those genes were screened to determine if any range/median, range/Q3, kurtosis or Q4/Q(2+3) slope calculation values were among the top 300 genes previously identified for each of the 4 calculations. The gene lists for each individual in the sample group were generated by (Mine Sample Extract) and imported into the STRING db to determine if any of the identified genes were associated with known Gene Ontology biological pathways. A detailed overview of the positional gene rank assignments for the 35 individuals is presented in Additional file 6 and summarized in Table 1 of the manuscript.

As predicted, the coordinated expression of multiple genes within a given pathway was accompanied by an increase in the number of Observed Gene Counts and by an increase in the positional ranking of these genes to higher gene-rank assignment locations. Using either unadjusted or MVA counts did not change the gene rank assignments when range/median, range/Q3, kurtosis and Q4/Q(2+3) slope calculations were used in the analysis (additional file 4, panels A vs. B). A summary of the positional rank analysis results presented in Table 1 confirm and extend the results presented Figure 3A of the manuscript.
