# Supplementary material for: Strategies for detecting and identifying biological signals amidst the variation commonly found in RNA sequencing data
Source: BMC Genomics. 2021 May 3;22:322. doi: 10.1186/s12864-021-07563-9 (PMC8091537; doi:10.1186/s12864-021-07563-9)
Supplement: Supplementary file 7 — Additional file 7. Rank-Ordered Distribution of White Blood Cells Among 35 Control Samples. [file 12864_2021_7563_MOESM7_ESM.docx]

**Additional file 7:**

**Rank-Ordered Distribution of White Blood Cells Among 35 Control Samples.**

The intraindividual gene ranking analysis presented in additional file 6 highlighted sample 33 with neutrophil and leukocyte enriched gene pathways. Therefore, we constructed a plot of the differential cell counts for our 35 samples to determine if either aberrant neutrophil or leukocyte cell numbers were contributing to this response. When the differential cell count data are plotted for each of the 35 samples, sample 33 clearly contained the largest number of WBC’s as depicted in panel A of Figure 1. When the differential cell counts are rank-ordered, the WBC and neutrophile counts in samples 6, 8 and 33 are shown to deviate from the majority of individuals within the sample group, as noted in panel B of Figure 1 and the removal of these three individuals significantly increased the R^2^ from 0.8249 to 0.9688 panel C of Figure 1. This analysis demonstrates that RNA extracted from individuals with WBC values that exceed 10 million WBC’s /ml of blood introduces undesired variability into the sequencing results. Furthermore, the trendline profiles of the differential cell count data suggests that the number of WBC’s within the various RNA extracts may contribute to the variability associated with the genes displaying linear trendline profiles. Normalizing RNA sequencing data in relation to the number of extracted cells may further reduce sample variance across the sample group as suggested in previous reports [16-19].


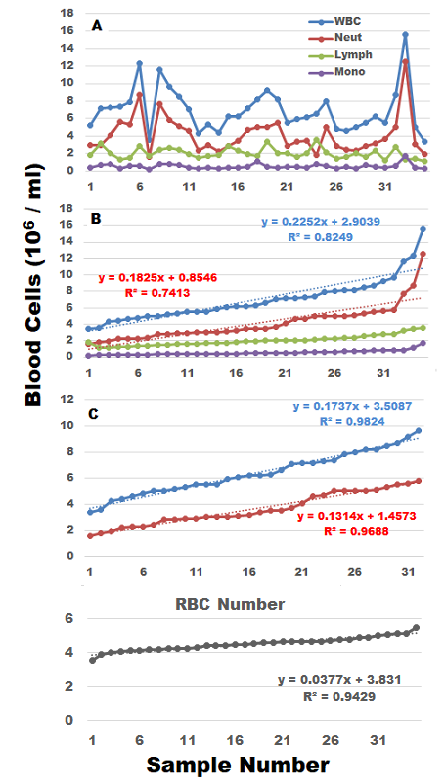


**Figure 4. Distribution of white blood cells in samples employed for RNA-seq analysis.** Panel A. The differential cell counts and total WBC counts for the 35 control samples are plotted in relation to sample number. Panel B. When WBC and differential cell counts are rank ordered, the increase in WBC’s is proportional to the increasing number of neutrophils in the blood. Panel C. Removal of samples 6, 8 and 33 from the analysis restores the linearity of the WBC count trendline among the remaining 32 samples. Differences in total WBC numbers increase biological variability and modify positional ranking results.
